# Metagenomic next-generation sequencing performed on blood samples for the early recognition of severe Pneumocystis pneumonia in critical hematological patients

**DOI:** 10.1097/MD.0000000000033399

**Published:** 2022-04-07

**Authors:** Xiang-Dong Shen, Xu-Dong Pan, Sen-Sen Shi, Ting Xu, Sheng-Li Xue, Jun Wang, Chao-Ling Wan, Yu-Ting Yao, Wei Lei, Tao Tao

**Affiliations:** a Department of Hematology, the First Affiliated Hospital of Soochow University, Suzhou, China; b Jiangsu Institute of Hematology, the First Affiliated Hospital of Soochow University, Suzhou, China; c National Clinical Research Center for Hematologic Diseases, Jiangsu Institute of Hematology, Suzhou, China; d Department of General Medicine, the First Affiliated Hospital of Soochow University, Suzhou, China; e Department of Pulmonary and Critical Care Medicine, the Affiliated Infectious Diseases Hospital of Soochow University, Suzhou, China; f Department of Pulmonary and Critical Care Medicine, the First Affiliated Hospital of Soochow University, Suzhou, China.

**Keywords:** early diagnose, hematology patients, metagenomic next-generation sequencing, peripheral blood, Pneumocystis pneumonia, severe pulmonary infections

## Abstract

Severe Pneumocystis pneumonia (PCP) has a poor prognosis, and its early and precise diagnosis is difficult in immunocompromised individuals. Therefore, this study explored the diagnostic value of metagenomic next-generation sequencing (mNGS) of peripheral blood in diagnosing severe PCP in patients with hematological diseases. This prospective study analyzed the clinical manifestations, mNGS results (from the peripheral blood), traditional pathogen detection results, laboratory test results, chest computed tomography (CT) images, treatments, and outcomes of severe PCP in hematological patients who were hospitalized in the 2 centers of the Affiliated Hospital of Soochow University between September 2019 and October 2021. A total of 31 cases of hematological diseases complicated with pulmonary infections, including 7 cases of severe PCP diagnosed by mNGS performed on peripheral blood samples, were analyzed. Traditional pathogen detection methods for PCP cannot be used. In contrast, the laboratory readings for *Pneumocystis jirovecii (Pj*) detected within 48 hours of symptom onset by mNGS on the 7 blood samples ranged from 12 to 5873, with a median value of 43. Under the guidance of the mNGS results, preemptive antimicrobial therapy with trimethoprim/sulfamethoxazole alone or in combination with caspofungin was administered to treat *Pj*. After treatment, 4 patients recovered, and 3 patients died of acute respiratory failure and acute respiratory distress syndrome (ARDS). MNGS performed on peripheral blood samples is optional but can provide early recognition of severe PCP and help guide empirical treatment in critical hematological patients.

## 1. Introduction

Pneumocystis pneumonia (PCP) is a common opportunistic infectious disease caused by *Pneumocystis jirovecii (Pj*) in both human immunodeficiency virus (HIV)-positive and HIV-negative patients.^[1]^ Currently, the number of PCP cases has grown in HIV-negative patients due to the increasing number of patients with hematological and solid malignancies, organ transplantations, hematopoietic stem cell transplantations (HSCTs), autoimmune disorders, and the administration of chemotherapy, immunosuppressive drugs, and glucocorticoid therapies.^[2,3]^ Previous studies have shown that severe PCP is one of the most common fatal infectious complications among hosts with hematological malignancies and HSCT recipients.^[4]^ Because of the nonspecific symptoms and signs of PCP, it is clinically characterized by delayed diagnosis, rapid progression to acute respiratory failure, coinfection with other pathogens, and high mortality in HIV-negative hosts (especially in patients with hematologic diseases).^[5,6]^ Therefore, early recognition and prompt initiation of anti*Pj* treatment are key factors for reducing mortality.

The gold standard for the diagnosis of PCP requires microscopic recognition of the structure of *Pj* from respiratory specimens such as sputum, bronchoalveolar lavage fluid (BALF), or lung tissue because *Pj* cannot be cultured.^[1]^ Unfortunately, the conventional smear method has some limitations, such as a low positive rate, time required, and difficulty in implementation in critical clinical settings.^[7]^ However, other conventional methods, including monoclonal antibody testing, immunofluorescence, and polymerase chain reaction, are also limited by their sensitivity, specificity, lack of standardization, and host heterogeneity.^[7]^ Due to the rapid progression of severe PCP, empirical treatment should be initiated as soon as suspected. However, traditional diagnostic approaches and invasive measures are difficult to implement in critically ill patients with hematological diseases because of dry cough, severe hypoxemia, multiple organ failure, coagulopathies, and low platelet counts. Consequently, there is an urgent need for a rapid and accurate pathogenic detection method to detect *Pj* in this group of severely ill patients for clinical use.

With the development of gene sequencing technology, high-throughput metagenomic next-generation sequencing (mNGS) technology has been used in the clinical diagnosis of infectious diseases.^[8,9]^ This innovative pathogen detection technology can directly detect the gene sequences of unknown pathogenic microorganisms in various samples, such as the blood, BALF, and cerebrospinal fluid.^[8–10]^ According to recent studies, mNGS of BALF is advantageous for diagnosing pathogens in pulmonary infectious diseases.^[11]^ However, the diagnostic sensitivity for pathogens in BALF is high and its specificity is low. Moreover, a large number of sick patients with severe blood disorders cannot tolerate bronchofibroscopy. To our knowledge, blood samples from patients are much easier to obtain in clinical practice because they contain fewer colonized and contaminating bacteria and have been used as the recommended specimens for pathogen detection in critically ill patients in several studies.^[12,13]^ It is still unclear whether blood samples can be used to detect *Pj* and provide evidence for the early diagnosis of severe PCP in patients with blood disorders; there are few studies in this area.

Herein, we report the clinical features of blood disease in patients with severe PCP to provide a reference for physicians for the early recognition of PCP. We also explored the clinical value of mNGS in peripheral blood to identify severe PCP in hematological patients. Finally, we provide data regarding the application value of mNGS in the diagnosis of PCP in future research.

## 2. Methods

### 2.1. Research subjects

This is a single center prospective observational study included a serial group of 31 patients with hematological malignancies who developed severe pneumonia. From September 2019 to October 2021, peripheral blood samples from 31 patients with symptoms of severe pneumonia after receiving immunosuppressive therapy or chemotherapy were sent for mNGS in the Department of Hematology of the First Affiliated Hospital of Soochow University and the Department of Pulmonary and Critical Care Medicine of the Affiliated Infectious Diseases Hospital of Soochow University. The inclusion criteria for this study were as follows: hematological patients with a history of receiving glucocorticoids (>4 weeks) and/or high-dose glucocorticoids (>1 mg/kg/d), chemotherapy, or other immunosuppressive drugs (>4 weeks)^[14]^; patients with severe pneumonia occurring after chemotherapy, those taking glucocorticoids or immunosuppressive agents, and those who did not receive prophylactic therapy for *Pj*; and the peripheral blood specimens used for testing were qualified, and those with ≥ 10 laboratory readings were included because *Pj* DNA could not be detected in the blood of healthy individuals. This study was conducted in accordance with the Declaration of Helsinki and approved by the Ethics Commission of the Affiliated Infectious Diseases Hospital of Soochow University. Written informed consent was obtained from all the patients.

### 2.2. Diagnostic criteria

All diagnoses of hematological patients were based on the World Health Organization criteria, and the primary diseases of this group were immune thrombocytopenic purpura, nonHodgkin lymphoma, myelodysplastic syndrome, and acute myeloid leukemia. Severe pneumonia was defined as clinical manifestations and/or auxiliary examinations with any of the following: dyspnea at rest, tachypnea at rest, persistent fever, and cough; arterial oxygen saturation (SaO_2_) of < 0.91 at rest, under ambient air; arterial oxygen tension of < 8.0 kPa (<60 mm Hg) at rest, under ambient air; and extensive interstitial shadowing with or without diffuse alveolar shadowing (“white out”) sparing the costophrenic angles and apices.^[15]^

### 2.3. Detection method (mNGS)

Based on high-throughput sequencing technology, the DNA sequence of the microorganism in the sample was obtained and compared with the microbial gene bank to identify pathogenic microorganisms. The detection process includes nucleic acid extraction, library construction, computer sequencing, bioinformatics analysis, and report interpretation.^[16]^ The detection process was based on Illumina NextSeq550Dx (Illumina) sequencing systems.

### 2.4. Data collection

In this single-arm prospective study, we analyzed the clinical course, methods of pathogen diagnosis, laboratory examinations, treatment, and outcomes of hematological patients with severe pneumonia. The following data were collected: sex, age, primary disease and treatment, epidemic history, pathogenic organism results, and antiinfection therapeutic regimen; clinical manifestations, symptoms, and signs, including cough, fever, shortness of breath, cyanosis, dyspnea, and results from general physical examinations; laboratory test results (performed within 24 hours of the occurrence of symptoms), including those obtained from routine blood tests, C-reactive protein levels, procalcitonin levels, coagulation function tests, sputum culture, throat swab culture, blood culture, (1,3)-β-D-glucan (BDG test), galactomannan (GM test), and chest computed tomography (CT), as well as outcome data.

### 2.5. Statistical methods

Software tools (SPSS, 25.0, IBM Corporation, Armonk, NY) were used to perform statistical descriptions and analyses, and MATLAB R2020a was used to draw graphs. Normally distributed measurement data are expressed as the mean ± standard deviation (*x* ± s). nonnormally distributed data are expressed as the median (upper and lower quartiles) [M (P_25_, P_75_)]. Enumeration data were expressed as percentages, and comparisons between groups were performed using the chi-square test and/or Fisher exact test. The significance level was set at α = 0.05, and the statistical significance was set at *P* < .05.

## 3. Results

### 3.1. Baseline characteristics

The Clinical manifestations, CT images, mNGS results, and criteria for severe pneumonia in all 31 patients were thoroughly examined by 3 specialists. A total of 7 hematological patients with PCP meeting the requirements were enrolled in our study (5 (71.43%) males and 2 (28.57%) females). The mean age of the patients was 40.4 years (range16–65 years). The underlying diseases included immune thrombocytopenic purpura in 1 case, non-Hodgkin lymphoma in 3 cases, myelodysplastic syndrome in 1 case, and acute myeloid leukemia in 2 cases. All patients with severe PCP had only received chemotherapy or were on immunosuppressive drugs, 4 patients had a medication history of glucocorticoids, and 3 patients received rituximab. Baseline patient data are summarized in Table 1.

**Table 1:** Baseline characteristics of PCP patients.

Number	Age (yr)	Gender	Comorbidity	Diagnosis	Treatment
1	16	Female	No	ITP	Methylprednisolone + Rituximab
2	46	Male	No	TCL	CHOPE + Chidamide
3	65	Male	No	DLBCL	Rituximab + CHOP
4	31	Female	No	MDS	Methylprednisolone + Rituximab
5	27	Male	No	GZL	FC + CAR-T
6	48	Male	Chronic liver disease	AML	Desitabine + Ara-C
7	50	Male	Hypertension	AML	Cladribine + CAG

AML = acute myeloid leukaemia, Ara-C = cytarabine, CAG = cytarabine+aclarithromycin+granulocyte colony stimulating factor, CAR-T = chimeric antigen receptor T-cell immunotherapy, CHOP = cyclophosphamide+epirubicin+vindesine+dexamethasone, CHOPE = cyclophosphamide+doxorubicin+vindesine+dexamethasone+etoposide, DLBCL = diffuse large B-cell lymphoma, FC = fludarabine+cyclophosphamide, GZL = gray zone lymphoma, ITP = immune thrombocytopenic purpura, MDS = myelodysplastic syndrome, PCP = Pneumocystis pneumonia, TCL = T-cell lymphoma.

### 3.2. Clinical manifestations and treatments

**Figure 1.**
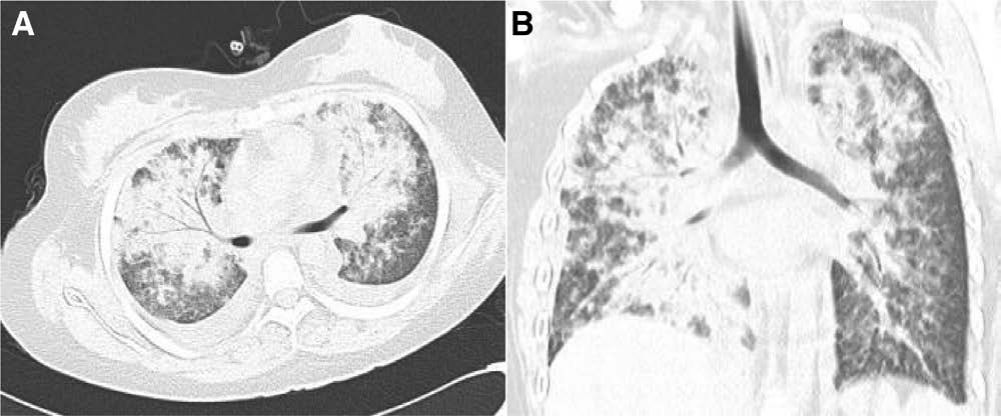
(A and B) Patient P1, female, 16 years old. Chest computed tomography (CT) showed multiple ground-glass opacities in the hilum to the periphery, consolidation, bronchial inflation signs, intralobular interstitial thickening and slight pleural effusion. The chest CT image was not reviewed because the patient died of ARDS. ARDS = acute respiratory distress syndrome.

Due to worsening hypoxia, 3 patients were admitted to the Department of Pulmonary and Critical Care Medicine, and the other 4 patients were admitted to the general wards. The main symptom in all 7 patients was fever (38.1–41.0°C). An intensifying dry cough was found in 4 patients; 4 patients had symptoms of rapid progressive dyspnea, and the oxygen saturation percentage was below 90% without oxygen inhalation. The signs in these patients, including heart rate and respiration rate, increased in 7 patients, and the breath sounds were vesicular over both lung fields in 3 patients. Arterial blood gas analysis showed respiratory failure in all patients: 4 patients received high-flow oxygen therapy, 3 patients received noninvasive ventilation, and 4 patients received methylprednisolone for respiratory failure and acute respiratory distress syndrome (ARDS). The 7 patients first received an empiric-combined antimicrobial therapy regimen, and then *Pj* was immediately targeted for coverage based on mNGS results. Two patients were treated with trimethoprim/sulfamethoxazole alone, and 5 patients were treated with trimethoprim/sulfamethoxazole combined with caspofungin. The 4 patients’ symptoms and chest CT changes resolved gradually after approximately 1 month of therapy. Unfortunately, 3 patients died of respiratory failure and ARDS despite combined therapy. The CT imaging findings were mainly bilateral ground-glass opacities, high-density shadows, and thickening of the interlobular septum. The chest CT findings of Patient 1 are shown in Figure 1. The patient died of ARDS (without repeat chest CT), and acute heart failure was ruled out. The clinical manifestations and treatments of all the patients are summarized in Table 2.

**Table 2:** Clinical symptoms, treatment and clinical outcomes of the 7 patients.

Number	Fever peak (°C)	Cough	Expectoration	Dyspnoea	Anti-*Pj* therapy	GC	Respiratory failure	Duration of therapy (d)	Outcome
1	40.0	+	+	+	TMP/SMX + CAS	+	+	20	Death
2	38.7	−	−	−	TMP/SMX + CAS	−	−	11	Survival
3	38.1	−	−	−	TMP/SMX	−	−	15	Survival
4	39.3	+	+	+	TMP/SMX + CAS	+	+	13	Death
5	39.8	−	−	−	TMP/SMX	−	−	21	Survival
6	40.0	+	−	+	TMP/SMX + CAS	−	−	12	Survival
7	38.6	+	+	+	TMP/SMX + CAS	+	+	29	Death

“+” means that the result exists, and “−” means that it does not.

CAS = caspofungin, GC = glucocorticoid, Pj = Pneumocystis jirovecii, TMP/SMX = trimethoprim/sulfamethoxazole.

### 3.3. PCP prophylaxis

To reduce the incidence and recurrence of PCP, prophylactic drugs for *Pj* should be used in patients with hematological malignancies.^[6,14]^ The first-line preventive medication regimen is as follows: 80 mg TMP/400 mg SMX or 160 mg TMP/800 mg SMX, once a day, or 160 mg TMP/800 mg SMX 3 times a week, and patients with allogeneic HSCT should continue treatment for PCP prevention for at least 6 months and immunosuppressive drugs.

### 3.4. Pathogen sequencing via mNGS

**Figure 2.**
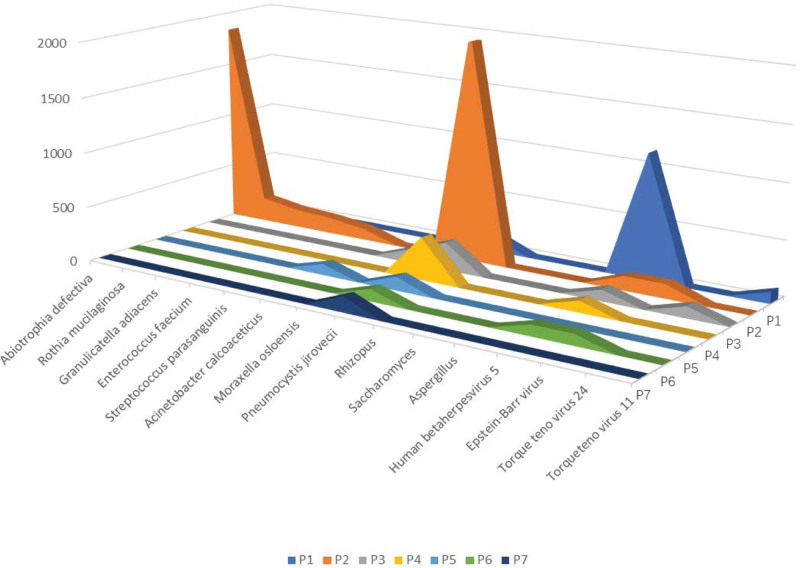
The ordinate numbers indicate the laboratory readings for pathogens from the mNGS results. Except for *Pneumocystis jirovecii, human beta herpesvirus 5* (cytomegalovirus) and Epstein–Barr virus, other pathogenic microorganisms are clinically considered to be background organisms. mNGS = metagenomic next-generation sequencing.

*Pj* was detected within 48 hours after the onset of respiratory symptoms by mNGS in the 7 blood samples, and the laboratory readings ranged from 12 to 5873; the median number of laboratory readings was 43 (Table 3). In our study, the mNGS results showed that 2 patients had *Pj* only, and 5 patients had 2 or more pathogenic microorganisms, implying a mixed infection diagnosed by experienced clinicians. Human cytomegalovirus (CMV) sequences were detected in 5 patients (71.43%). No other potential pathogens causing pneumonia were identified. The laboratory readings of *Pj* in the deceased patients were 43, 388, and 42, and those in the surviving patients were 5873, 253, 25, and 12, respectively. Although the laboratory readings of *Pj* in patient No. 2 (5873 laboratory readings) were the highest among all patients, the patient clinical symptoms were not obviously different from those of the other patients. The distribution and abundance of microorganisms, according to the mNGS results, are shown in Figure 2.

**Table 3:** Pathogens detected by mNGS and traditional pathogen detection methods.

Number	Coinfection by mNGS	Reads of *Pj* by mNGS	Reads of CMV by mNGS	Bacterial cultures from sputum or throat swab	BDG test (pg/mL, normal range 0–70)	GM test (pg/mL, normal range 0–0.5)	CRP (mg/L, normal range 0–6)	PCT (ng/mL, normal range 0–0.5)
1	*Pj*, CMV	43	59	–	>600	<0.25	>15.36	0.22
2	*Pj*, CMV EBV	5873	23	–	151.3	0.11	>15.36	0.13
3	*Pj*, CMV	253	77	–	<10	0.51	>15.36	0.096
4	*Pj*, CMV	388	99	–	243	0.13	>15.36	0.162
5	*Pj*	25	-	–	<10	0.09	>15.36	0.139
6	*Pj*, CMV	12	26	Bc	<10	0.13	>15.36	0.373
7	*Pj*	42	–	–	37.5	0.25	>15.36	0.18

Bc = Burkholderia cenocepacia, BDG test = 1-3- β- D glucan test, CMV = cytomegalovirus, CRP = C-reactive protein, EBV = Epstein–Barr virus, GM test = galactomannan test, mNGS = metagenomic next-generation sequencing, PCT = procalcitonin, PJ = *Pneumocystis jirovecii*.

### 3.5. Analysis of traditional pathogenic tests

**Figure 3.**
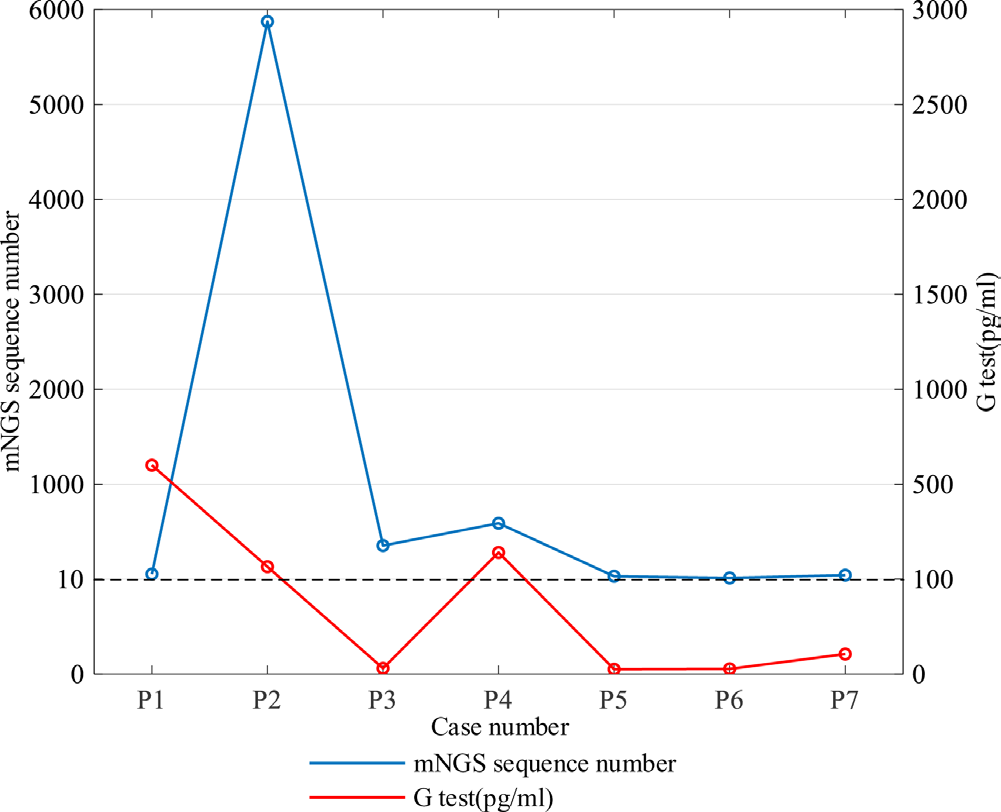
The coordinate points above “----” indicate positive results, while the coordinate points below indicate negative results.

In this study, samples from 7 patients were sent for blood culture (aerobic and anaerobic bacteria), sputum culture, throat swab culture, BDG test, and GM test simultaneously. Blood cultures from all 7 patients were negative, and throat swab cultures from 1 patient revealed *Burkholderia cenocepacia*. Among them, 3 patients had positive BDG test results (>600, 243, and 151.3 pg/mL), and 4 patients had negative BDG test results (<70 pg/mL). The correlation between the BDG test and laboratory readings of *Pj* via mNGS is plotted as a line in Figure 3. All GM test results were negative in this group of patients. The C-reactive protein levels in this group increased to varying degrees, whereas the procalcitonin levels were normal. A comparison of the mNGS results and conventional tests is shown in Table 3.

### 3.6. Results of laboratory indicators

The lactate dehydrogenase (LDH) level of these patients was 346.1 U/L (223.1–842.5), which was higher than the reference value (120–250 U/L). The albumin level was 27.11 ± 2.77 g/L lower than the reference value (40–55 g/L). The other indicators were almost normal, and the results of the routine blood, coagulation, and biochemical analyses are shown in Table 4.

**Table 4:** Laboratory tests from the 7 patients.

Indicator	Normal range	Results
Blood routine		
White blood cell count, ×10^9^/L	[3.5, 9.5]	5.89 ± 6.52
Neutrophil count, ×10^9^/L	[1.8, 6.3]	5.14 ± 6.07
Hemoglobin, g/L	[115, 150]	76.71 ± 26.39
Platelet, ×10^9^/L	[125, 350]	102.43 ± 114.52
Lymphocyte count, ×10^9^/L	[1.1, 3.2]	0.57 ± 0.38
Lymphocyte (%)	[20, 50]	11.85 ± 28.42
Coagulation		
PT (s)	[11.5, 15.5]	13.49 ± 1.12
APTT (s)	[26, 40]	16.9 ± 16.45
TT (s)	[14, 21]	24.74 ± 11.5
Fbg (g/L)	[2, 4]	10.24 ± 7.27
PT-INR	[1.0, 2.0]	2.7 ± 2.5
Blood biochemistry		
TP (g/L)	[65, 85]	56.81 ± 8.49
ALB (g/L)	[40, 55]	27.11 ± 2.77
LDH (U/L)	[120, 250]	346.1 [223.1, 842.5]
ALT (U/L)	[7, 40]	35.69 ± 19.7
AST (U/L)	[13, 35]	29.80 ± 15.28
Cr (μmol/L)	–	54.76 ± 17.71
GLU (mmol/L)	[3.9, 6.1]	5.08 ± 1.20
Na (mmol/L)	[137, 147]	137.76 ± 4.34
K (mmol/L)	[3.5, 5.3]	3.74 ± 0.58
Ca (mmol/L)	[2.11, 2.52]	1.98 ± 0.13

ALB = albumin, ALT = alanine transaminase, APTT = activated partial thromboplastin time, AST = aspartate aminotransferase, Ca = serum calcium, Cr = creatinine, Fbg = fibrinogen, GLU = blood glucose, K = serum potassium, LDH = lactic dehydrogenase, Na = serum sodium, PT = prothrombin time, PT-INR = prothrombin time intertional normalized ratio, TP = total protein, TT = thrombin time.

## 4. Discussion

An epidemiological study showed that *Pj* is an opportunistic pathogen that is transmitted through the respiratory tract in the form of aerosols and exists in alveolar epithelial cells to colonize normal immune hosts.^[17,18]^ During normal immune function, *Pj* is cleared through immune processes. In contrast, PCP may occur when the immune function of patients is compromised.^[17,18]^ The common clinical symptoms are fever, cough, and dyspnea. If expectoration is present, co-infection with other bacteria should be suspected. Rapid progression of dyspnea, hypoxemia, and pulmonary imaging indicates the development of acute respiratory failure and ARDS.^[5,15,19]^ Patients with hematologic diseases are prone to PCP, which is characterized by rapid progression, easy misdiagnosis, difficult diagnosis, and high mortality.^[2,5,20]^ This study reported the clinical features of 7 hematological patients with severe pneumonia who were diagnosed with severe PCP (with or without other mixed pathogen infections) using mNGS of blood samples. The early clinical manifestations of these 7 patients with critical PCP were nonspecific. Uncontrolled fever after an empirical combination of antiinfective therapy was the presenting symptom, which indicated that the patients probably had a *Pj*-related bloodstream infection. Therefore, if patients with severe pulmonary infections cannot provide high-quality respiratory specimens for microbiological testing, preemptive hematological mNGS should be proposed because the positivity rate is usually high in these patients. The main clinical presentations of these patients and their poor prognoses in our study are consistent with those in previous studies.^[2,5,20]^ Our research demonstrates that the early stages of PCP in patients with blood disease tend to be misdiagnosed; thus, clinicians must be aware of the illness and keep it in mind.

Previous studies have suggested that specimens from the respiratory tract, such as BALF and sputum, are preferred for pulmonary pathogen detection. However, it is worth noting that respiratory tract specimens often contain colonized bacteria and have low specificity for diagnosing pulmonary infections.^[11,21]^ The accurate identification of causative pathogens using mNGS is a challenge for physicians. Furthermore, sputum collection and bronchofibroscopy cannot be performed in hematological patients with severe PCP. A few studies have reported that mNGS performed on blood samples is superior to mNGS performed on respiratory specimens for diagnosing PCP in immunosuppressed populations.^[22–24]^ Similarly, our study is the first to report the application of mNGS to blood samples for early identification of severe PCP in patients with hematologic diseases. The results of 5 patients suggested that *Pj* was associated with CMV reactivation, and 3 patients recovered after timely anti*Pj* and anti-CMV treatments. Researchers from Turkey have found that coinfections with CMV and ARDS are risk factors for the survival of patients with severe PCP.^[25]^ Hence, during combination therapy for severe PCP, screening and targeted treatment of CMV are equally important. In addition, 2 patients had the *Pj* sequence only (without evidence of other pathogens). Among them, 1 patient improved after anti*Pj* treatment alone and the other patient died of acute respiratory failure. Therefore, our findings suggest that mNGS performed on blood specimens has potential value in the early diagnosis of PCP and in guiding antiinfection protocols, which is consistent with previous studies.^[22–24]^ The pathological process of severe PCP is due to the decline in immune T cell function, which leads to the transformation of *Pj* from a latent to a pathogenic state in alveolar epithelial cells, resulting in excessive immune damage and respiratory dysfunction.^[18,19]^ In this study, the blood cultures of all the patients were negative. In contrast, a certain amount of *Pj* was detected through mNGS of blood samples from hematological patients with severe PCP. The mechanism by which *Pj* enters the circulation from the latent state requires further study.

A positive result on the BDG test is nonspecific and may indicate other fungal infections, as seen in some blood disease patients without fungal infections. Studies have shown that the sensitivity and specificity of the BDG test for PCP are 94.8% and 86.3%, respectively, and a negative result can rule out PCP.^[26]^ Among the 7 patients in this study, the BDG test was positive in 3. The levels in the other 4 patients were <10 pg/mL, which showed differences compared with the mNGS technology. Studies have shown that LDH level, lymphocyte count, and albumin level may partially indicate the prognosis of severe PCP.^[4,19,27]^ LDH levels reflect the degree of lung injury to a certain extent. An LDH level of >500 U/L may be associated with acute lung injury. The LDH levels of the 2 deceased patients were 661.5 U/L and 842.5 U/L, but the LDH level of the 4 patients who experienced improvements was <500 U/L, consistent with previous reports.

This study has a few limitations. First, this was a retrospective study with a small sample size, conducted in only 2 centers. The mNGS detection of *Pj* has not been verified using traditional diagnostic methods such as blood smears, monoclonal antibody tests, or polymerase chain reaction. Second, mNGS performed on blood specimens was not compared with mNGS performed on respiratory specimens for the diagnosis of PCP. In addition, it remains unclear how many laboratory readings of *Pj* are necessary to diagnose PCP. Further research will be conducted to address these deficiencies.

## 5. Conclusions

In summary, patients with hematological malignancies are at a high risk of rapidly developing severe PCP with atypical clinical symptoms. Fever, dry cough, and progressive dyspnea are the main symptoms that indicate the occurrence of acute respiratory failure and ARDS. mNGS performed on peripheral blood samples is an option but can provide early recognition of severe PCP and help guide empirical treatment in critically ill hematological patients.

## Author contributions

**Data curation:** Xiang-Dong Shen, Jun Wang, Chao-Ling Wan.

**Formal analysis:** Sen-Sen Shi, Chao-Ling Wan, Yu-Ting Yao.

**Funding acquisition:** Tao Tao.

**Methodology:** Ting Xu, Sheng-Li Xue, Jun Wang.

**Software:** Chao-Ling Wan.

**Supervision:** Tao Tao, Sheng-Li Xue.

**Writing – original draft:** Xiang-Dong Shen.

**Writing – review & editing:** Xu-Dong Pan, Wei Lei.
